# The effect of flue-curing procedure on the dynamic change of microbial diversity of tobaccos

**DOI:** 10.1038/s41598-021-84875-6

**Published:** 2021-03-08

**Authors:** Binbin Hu, Kaiyuan Gu, Jiangshiqi Gong, Ke Zhang, Dan Chen, Xian He, Yi Chen, Kaixian Gao, Yan Jin, Kun Huang, Yanmei Zhu, Congming Zou

**Affiliations:** 1 Yunnan Academy of Tobacco Agricultural Sciences, Kunming, 650021 Yunnan China; 2 Wenshan Municipal Tobacco Company, Wenshan, 663000 Yunnan China

**Keywords:** Ecology, Microbiology, Plant sciences

## Abstract

The purpose of the study is to explore the effect of flue-curing procedure on the diversity of microbial communities in tobaccos and the dynamic change of compositions of microbial communities in the flue-curing process. It expects to provide a theoretical basis for the application of microbes in tobacco leaves and a theoretical basis and idea for optimization of the flue-curing technologies. By investigating tobacco variety K326, the tests were carried out for comparing the conventional flue-curing procedure and dry-ball temperature set and wet-ball temperature degradation flue-curing procedure. Based on the culture-independent approach and high-throughput sequencing procedure, the relationship between the flue-curing procedure for tobaccos and microbial communities in tobaccos was revealed by measuring the dynamic change of microbial communities. The results indicated that:(1) Relative to surface wiping method, washing method was more suitable for the sampling of microbes on the surface of tobacco leaves; (2) Dry-ball temperature set and wet-ball temperature degradation flue-curing procedure was more favorable for maintaining the microbial diversity of tobaccos; (3) Relative to bacteria of the tobaccos, the succession rule of the fungal communities in tobaccos was relatively steady; (4)Compared with bacterial community diversity, the fungal community diversity presented an obvious negative correlation with temperature and humidity during the flue-curing process. (5) The function of bacterial communities in tobaccos matched with the material transformation law of tobaccos, having a direct correlation on the flue-curing process. In short, Dry-ball temperature set and wet-ball temperature degradation flue-curing procedure can more favorably maintain the microbial diversity of tobaccos; moreover, the function of the tobacco system involved in microbes in tobaccos was closely related to the material transformation law of tobaccos in the flue-curing process. It validated that the bacteria in tobaccos play an important role in the flue-curing process of tobaccos.

## Introduction

Microbial community influences the whole growth stage of tobaccos and later flue-curing process of tobaccos, which plays an important role in formation of tobacco quality^1^. In the growth process of tobaccos, a large number of secondary metabolites are excreted or exuded from the root system and leaves^2,3^. These metabolites provide sufficient nutrition for the growth of various microbes and form a special microenvironment harboring numerous microbes on the surface and inside the matured and harvested tobacco leaves^4^. Microbes play an important role in many aspects, including shortening the fermentation time, controlling the harmful components (such as nicotine, protein and tobacco-specific nitrosamines (TSNAs) in tobacco leaves and enhancing the aroma of tobacco leaves, thus improving the tobacco quality^5–7^. Nevertheless,existing research mainly concentrates on the aging and curing period of tobacco leaves^8–11^; however, the microbial diversity and its dynamic change law in the flue-curing period are not well researched. In existing researches, the microbial communities on the surface of flue-cured tobacco leaves and their dynamic change are mainly focused onculture method or traditional primary means^12,13^. Traditional means for microbial isolation and identification show limitations when mining the information on microbes in the environment, through which less information is acquired and some unculturable microbes are hard to be isolated and identified^14^; with the development of the second-generation sequencing procedure, high-throughput sequencing method presents more significant superiorities compared with Sanger sequencing method: firstly, a great number of parallel samples can be synchronously measured by utilizing ChIP sequencing; secondly, the times of measuring DNA sequences can reflect the abundance of the DNA fragment, showing the quantitative function; thirdly, single sequencing reaction is able to analyze millions of samples at a low cost and exhibits a powerful sequencing ability, which is incomparable for traditional sequencers^15^. Recently, the IonS5XL high-throughput sequencing techniques were used toreveal the fungal community of the petioles and lamina of tobacco leaves infectedwith pole rot during flue-curing. The results demonstrates that both culturablefungal diversity and fungal sequence diversity was higher at stem-drying stage than theyellowing and color-fixing stages, and diversity was higher with leaf lamina than petiolesrevealing that the changes in fungal composition and diversity during the curing processwere similar with both methods^16^.

The conventional flue-curing procedure is easy to operate that is the most widely used at present, but there are some flaws in the conventional flue-curing process, such as high likelihood of yellow tobacco leaves and weaker aroma of cured tobacco leaves, which reduce the industrial applicability of the cured tobacco leaves and cause losses to tobacco farmers. When meeting the aberrant climate, the conventional flue-curing procedure always raises the ratio of worthless flue-cured tobacco.As the state-of-the-art control procedur for tobaccos, temperature- and humidity-controlled procedure improves the temperature and humidity control fields not involved in common curing procedure^17–19^. It greatly enhances the quality of flue-cured tobacco leaves and is possibly related to the microbial diversity of tobacco leaves and its dynamic change in the procedure^18,20,21^. In this study, the tests were performed for comparing conventional flue-curing procedure and the dry-ball temperature set and wet-ball temperature degradation flue-curing procedure. Based on culture-independent method and high-throughput sequencing procedure, the effect of the flue-curing procedure on, and the dynamic change of, the microbial diversity of tobaccos were revealed by measuring the dynamic change of microbial communities; furthermore, the mechanism of increasing the quality of tobacco leaves by microbes in the flue-curing stage was analyzed. It provides a theoretical basis for the application of microbes in tobacco leaves and a new evaluation index for the maturity of flue-cured tobacco leaves, expecting to finally further improve the flue-curing procedure for tobaccos.

## Materials and methods

### Test materials

The test was conducted on the Yanhe test base in Yuxi, Yunnan Province, China from April, 2019 to October, 2019. K326 variety provided by Yuxi Tobacco Seed Co. LTD was used for the test. The moderately matured fresh tobacco leaves taken from the middle part of plants were selected for test analysis. Based on standard harvest and rod-weaving methods of tobaccos, the distribution of tobacco leaves was ensured to be consistent and tobacco leaves were flue-cured in roasters produced inYanhe research base.

### Test design

**Figure 1:**
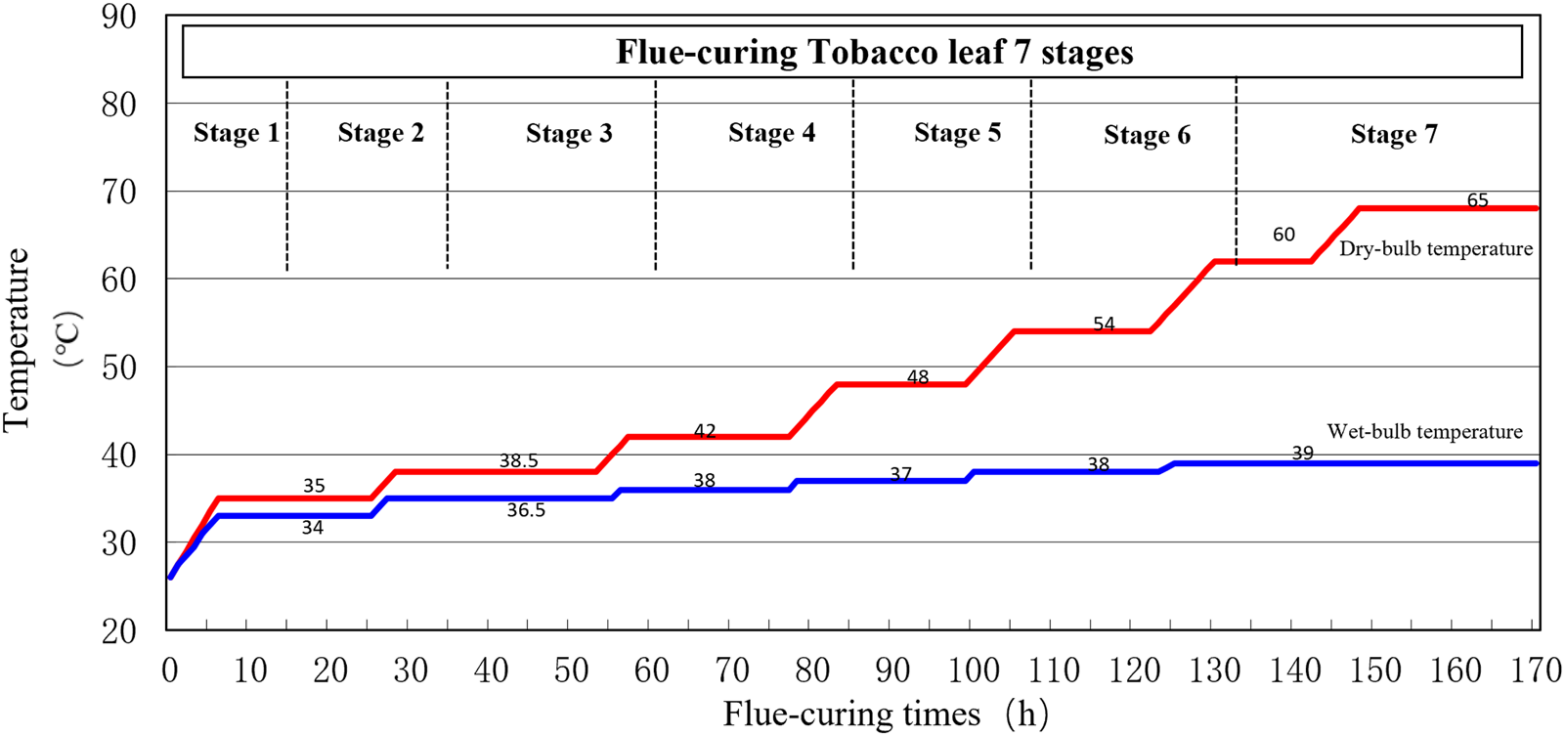
Conventional flue-curing procedure (CT) used in the bulk curing barns in Yuxi tobacco-growing area.

**Figure 2:**
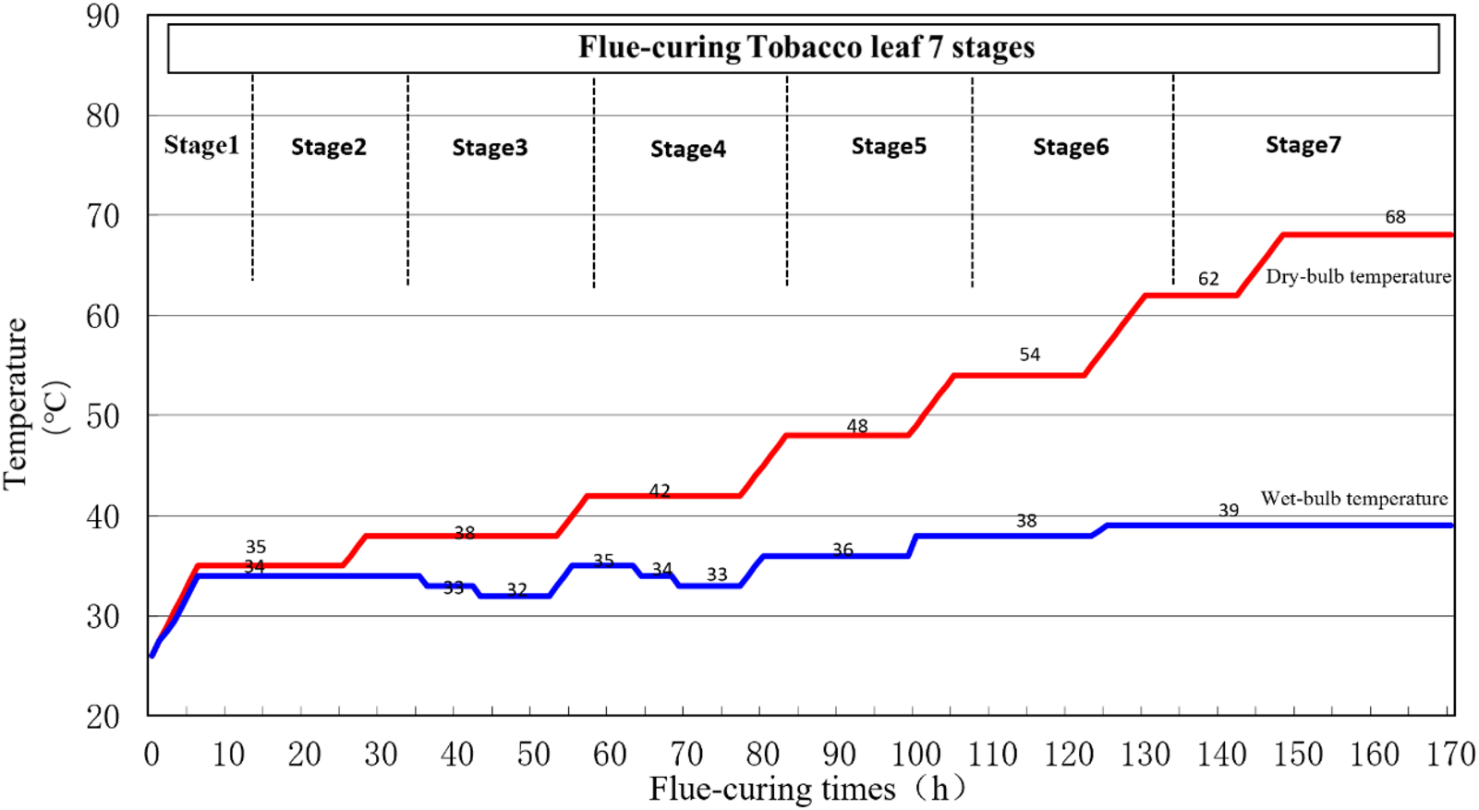
Dry-ball temperature set and wet-ball temperature degradation flue-curing procedure (DSWD) used in bulk curing barns in Yuxi tobacco-growing area.

Two flue-curing procedures were selected according to the requirements for flue-curing procedure, involving (1) conventional flue-curing procedure (Fig. 1) and (2) dry-ball temperature set and wet-ball temperature degradation flue-curing procedure (Fig. 2). Seven stages flue-curing process was adopted and the dry-bulb temperature and wet-bulb temperature were gradually increased during the flue-curing of tobacco. For conventional flue-curing procedure, the dry-bulb temperature and wet-bulb temperature werestationarity at each stage. The dry-bulb temperature was gradually rose from 35 to 65 ℃ and the wet-bulb temperature was gradually rose from 34 to 39 ℃. For dry-ball temperature set and wet-ball temperature degradation flue-curing procedure, the wet-ball temperature was gradually fell to 32 ℃ from 34 ℃ at third stage and the wet-ball temperature was continuously fell to 33 ℃ from 35 ℃ at fourth stage. As for other stages, the dry-bulb temperature and wet-bulb temperature were stationarity^22^.

### Sampling method

Flue-curing procedure and the temperature- and humidity-controlled procedure were synchronously implemented in the whole flue-curing process. Sampling was performed at a same dry-bulb temperature point. Three random tobacco leaves were sampled for every temperature point. The tobacco leaf samples in the flue-curing process were taken and stored in the sterile environment; moreover, the microbes in tobaccos were timely sampled. The samples were sampled at six time point and the original fresh tobacco leaves were the same for different flue-curing procedures. When considering the flue-curing procedures (CT and DSWD), microorganism types (bacteria and fungus), microorganism locations (surface and endophytic), sampling times (six time points) and duplicate samples, there are 88 samples were sampled during experiments.

The sampling method for microbes on the surface of leaves: (1) the leaves were rotationally wiped by using swabs (each leaf was wiped by utilizing 1–3 swabs depending on the size thereof); (2) the explored objects were placed in a sterilized container, in which 30 ml phosphate buffer saline (PBS, 145.45 mM NaCl, 15.50 mM Na_2_HPO_4_, 2.50 mM NaH_2_PO_4_, pH 7.2) solution was added, followed by vibration. In this way, microbes were fully exfoliated from the surface of the objects and gathered in the PBS solution. DNA extraction was conducted after the buffer solution was filtered by applying the filter membrane.

Sampling method for endophytes: leaves were washed with water and then a glass bead sterilizer was violently shaken in bacteria free water in order to physically remove bacteria (removing the disturbance of microbes on the surface of leaves as far as possible). The leaves, on which microbes were removed, were stored at − 80 ℃.

### Genome extraction, amplification and sequencing

DNAs in genomes of samples were extracted by employing cetyltrimethylammonium bromide (CTAB) method. The 1000 μl CTAB, 20 μl lysozyme and sample were mixed in the tube and incubated in water bath at 65 ℃ for 2 h. The solulation was mixed every 0.5 h during the incubation. After centrifugation, the 950 μl supernatant was mixed with 950 μl phenol-chloroform-isoamylol (25:24:1) and centrifugated at 12,000 rpm for 10 min. The supernatant was mixed with equal volume chloroform-isoamylol (24:1) and centrifugated at 12,000 rpm for 10 min. The supernatant was mixed with 3/4 volume isopropanolandprecipitated at − 20 ℃ for 10 h. After centrifugated at 12,000 rpm for 10 min, the supernatant was removed and the precipitation was washed by 1 ml ethanol for two times. The precipitation was dried at room temperature and dissolved by 50 μl ddH_2_O. The DNA solution was mixed with 1 μl RNase at 37 ℃ for 15 min. Afterwards, the purity and concentration of DNAs were tested by using agarose gel electrophoresis; a proper quantity of sample DNAs were taken into a centrifuge tube and then diluted to 1 ng/μl by applying bacteria free water.

By taking the diluted genomic DNAs as the template, polymerase chain reaction (PCR) was performed by employing specific primers with Barcode. The 16S and ITS PCR reactions were carried out as pervious literatures^16,23^.

Corresponding zones of primers: the primers (515F and 806R) in 16S V4 zone were used to identify bacterial diversity. The 515F sequence (5′ to 3′): GTGCCAGCMGCCGCGGTAA. The 806R sequence (5′ to 3′): GGACTACHVGGGTWTCTAAT.

Primers (ITS1-1F-F and ITS1-1F-R) in the ITS-1F zone were applied to identify the fungal diversity. The ITS1-1F-F sequence (5′ to 3′): CTTGGTCATTTAGAGGAAGTAA. The ITS1-1F-R sequence (5′ to 3′): GCTGCGTTCTTCATCGATGC.

Sequencing libraries were generated using Illumina TruSeq DNA PCR-Free LibraryPreparation Kit (Illumina, CA, USA) following manufacturer’s recommendations and index codeswere added. The library quality was assessed on the Qubit@ 2.0 Fluorometer (Thermo Scientific, MA, USA)and Agilent Bioanalyzer 2100 system (CA, USA). At last, the library was sequenced on an IlluminaNovaSeq platform and 250 bp paired-end reads were generated.

### Analysis of sequencing information

#### Processing of sequencing data

According to Barcode sequences and primer sequences for PCR amplification, the data on various samples were separated from the offline data. After intercepting Barcode and primer sequences, reads of each sample were assembled by using FLASH (V1.2.7, http://ccb.jhu.edu/software/FLASH/)^24^. On this basis, the attained assembly sequence was considered as the original Tags data (raw Tags); the raw Tags obtained through assembly needed to be rigorously filtered so as to obtain high-quality Tags (clean Tags). By referring to Tags quality control flow of Qiime (V1.9.1, http://qiime.org/scripts/split_libraries_fastq.html)^25^, the following operations were performed: (a) Tags interception: the raw Tags were intercepted at the site of the first low-quality base where the number of bases with continuous low-quality scores (default threshold of quality not larger than 19) reached the preset length (default length being as 3); (b) filtering of Tags length: the Tags with the length of continuous high-quality bases lower than 75% of the Tags length were further filtered out from the Tags dataset after interception. Tags obtained through the above processing needed to remove the chimeric sequences. The chimeric sequences in Tags sequences were aligned and detected through https://github.com/torognes/vsearch/ and the database of annotations of species. Eventually, the chimeric sequences were removed to attain the final valid data (effective Tags).

The Sequencing data for bacterial and fungal communities were deposited in the National Center for Biotechnology Information (NCBI)Sequence Read Archive (http://trace.ncbi.nlm.nih.gov/Traces/sra/) under the accession numbers of PRJNA685271.

#### OTU clustering and species annotation

All effective Tags of all samples were clustered by applying Uparse software (Uparse v7.0.1001, http://www.drive5.com/uparse/). The sequences were clustered into operational taxonomic units (OTUs) based on 97% of identity^26^; moreover, it was necessary to select the representative sequence of OTUs: according to the algorithm principle, the sequence in OTUs with the highest occurrence frequency was screened as the representative sequence thereof. The species annotation was performed on the OTUs sequences. Moreover, the bacterial species annotation (setting the threshold in the range of 0.8–1) was analyzed by applying Mothur method and SSUrRNA database of SILVA132 (http://www.arb-silva.de/). For fungal species annotation, the Unit (v7.2) database was adopted.Thus, the taxonomic information was acquired and the community compositions of various samples were separately computed at different classification levels, e.g. kingdom, phylum, class, order, family, genus and species. Multiple sequences were rapidly compared by applying MUSCLE software (Version 3.8.31, http://www.drive5.com/muscle/) to attain the phylogenetic relationships of all representative sequences of OTUs.Finally, the data of various samples were normalized to the sample with the smallest data size.

#### Correlation analysis of environmental factors

When performing Spearman correlation analysis, Spearman correlation coefficients of species and environmental factors were first calculated by applying corr.test function in psych package of R software and also their significance was tested. Subsequently, visualization was achieved by using pheatmap function in pheatmap package.

Mantel test was undertaken by using R vegan package. According to species matrix and the provided data matrix of environmental factors, the distance matrices of two types of data were first transformed by utilizing vegdist function; afterwards, the Spearman correlation analysis of the two types of matrices was performed based on mantel function to attain r and P values.

#### Function annotation

Tax4Fun function prediction was realized by using the nearest neighbor algorithm based on the minimum similarity of 16S rRNA sequences. To be specific, the 16S rRNA gene sequence in the complete genomes of prokaryotes in KEGG database was extracted and aligned with the SILVA SSU Ref NR database (BLAST bitscore > 1500) by using BLASTN algorithm to establish the correlation matrix. The function information of the complete genomes of prokaryotes in the KEGG database annotated through UProC and PAUDA methods was mapped into SILVA database to realize the function annotation of the SILVA database. By taking the sequences in the SILVA database as the reference, the sequenced samples were clustered into OTUs, thus further attaining the function annotation information.

Based on the support of published literatures, the ecological functions of fungi were classified and the FunGuild database was builded. The functions were annotated by referring the FunGuild database.

## Results and analysis

### Comparison of sampling methods for microbes on the surface of tobacco leaves

According to previous research, two sampling methods for microbes on the surface of tobaccos were selected to separately perform extraction and amplification of genome DNAs after sampling the microbes. As shown in Table 1, as for the first sampling method, the DNAs extracted from two tobacco leaf samples (fresh tobacco leaves and tobacco leaves in the later yellowing period) were both unqualified after subjected to amplification. Therefore, the first method was not suitable for extracting the microbes on the surface of tobacco leaves. The two tobacco samples extracted by using the second method both allowed favorably amplification and their amplification results were both proper. Thus, the second method was applied to sample the microbes on the surface of tobacco leaves subsequently.

**Table 1:** Comparison of effects of the two methods for sampling microbes on the surface of tobaccos.

Samples	Sequence characterized amplified region	Test result	Test conclusion
Surface 1 of fresh tobacco leaves	S5-16SV4	Unqualified	Unsuitable
Surface 2 of fresh tobacco leaves	S5-16SV4	Qualified	Suitable
Surface 1 of tobacco leaves in the later yellowing period	S5-16SV4	Unqualified	Unsuitable
Surface 2 of tobacco leaves in the later yellowing period	S5-16SV4	Qualified	Suitable

1 and 2 represent the first and second methods for sampling microbes on the surface of tobaccos, respectively.

### OTU clustering analysis

To explore the species compositions of various samples, OTUs clustering was carried out on effective Tags of all samples based on 97% of identity; afterwards, species annotation was performed on the OTUs sequences. According to OTUs results obtained through clustering and research requirements, the common and specific OTUs among different samples (groups) were analyzed.

#### OTU clustering analysis of bacteria in tobacco leaves

**Figure 3:**
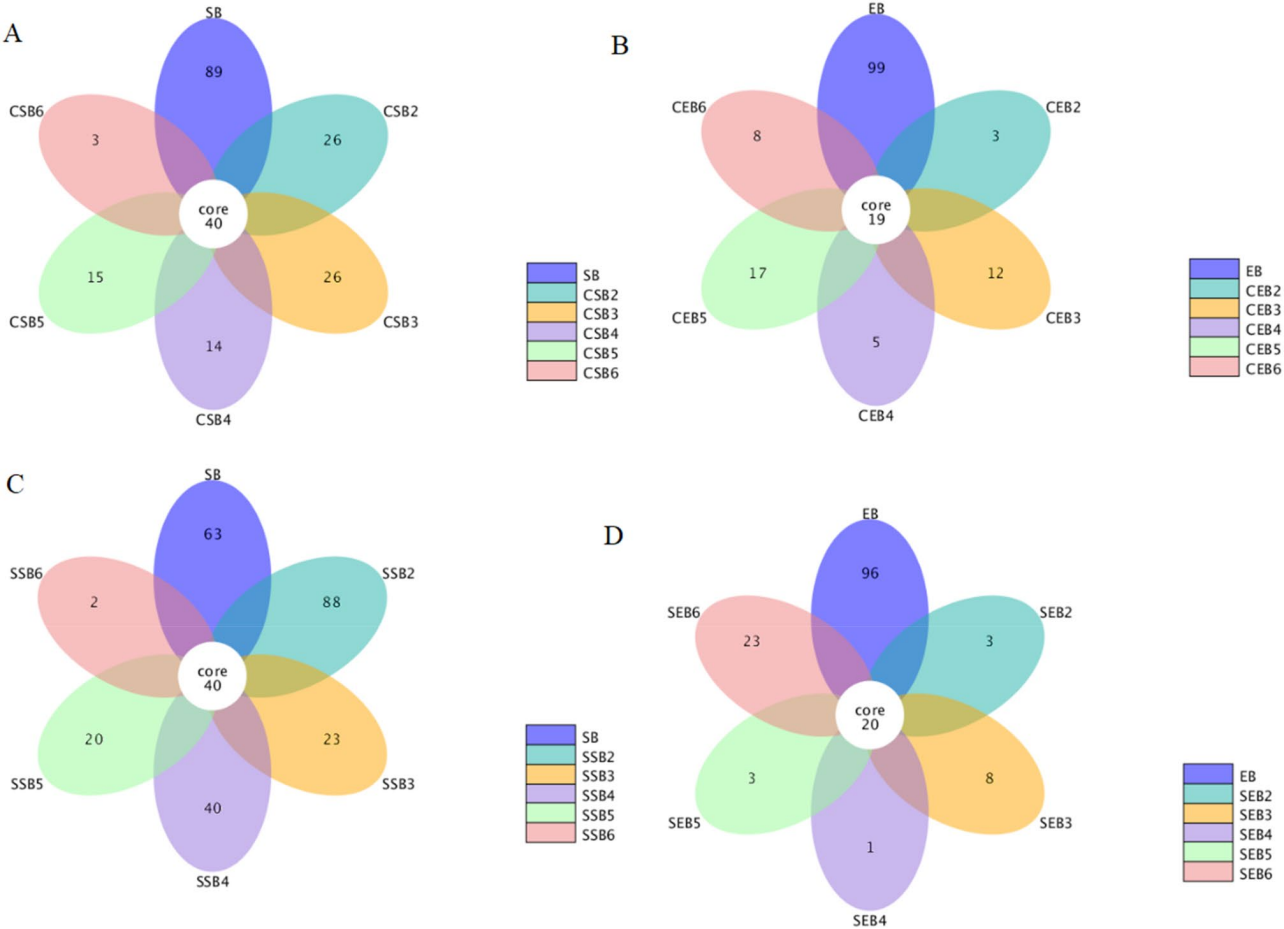
Petal diagrams of OTUs in samples flue-cured through conventional procedure and temperature- and humidity-controlled procedure under different sampling stages (SB: the surface bacteria of fresh tobacco leaves; EB: endophytic bacteria of fresh tobacco leaves; CSB: the surface bacteria of tobacco leaves flue-cured using conventional procedure; CEB: endophytic bacteria of tobacco leaves flue-cured using conventional procedure; SSB: the surface bacteria of tobacco leaves flue-cured using temperature- and humidity-controlled procedure; SEB: endophytic bacteria of tobacco leaves flue-cured using temperature- and humidity-controlled procedure; 2–6 represent different sampling stages).

The result is shown in Fig. 3. Each petal in the petal diagram represents a group (sample) and different colors mean diverse samples (groups); the number at the core stands for the total number of OTUs in all samples; the number in each petal denotes the number of OTUs specific in the sample (group). It can be seen from the figure that the numbers of the core microbial communities subjected to conventional flue-curing procedure and dry-ball temperature set and wet-ball temperature degradation flue-curing procedure were basically consistent, showing no great change.

#### OTU clustering analysis of fungi in tobacco leaves

**Figure 4:**
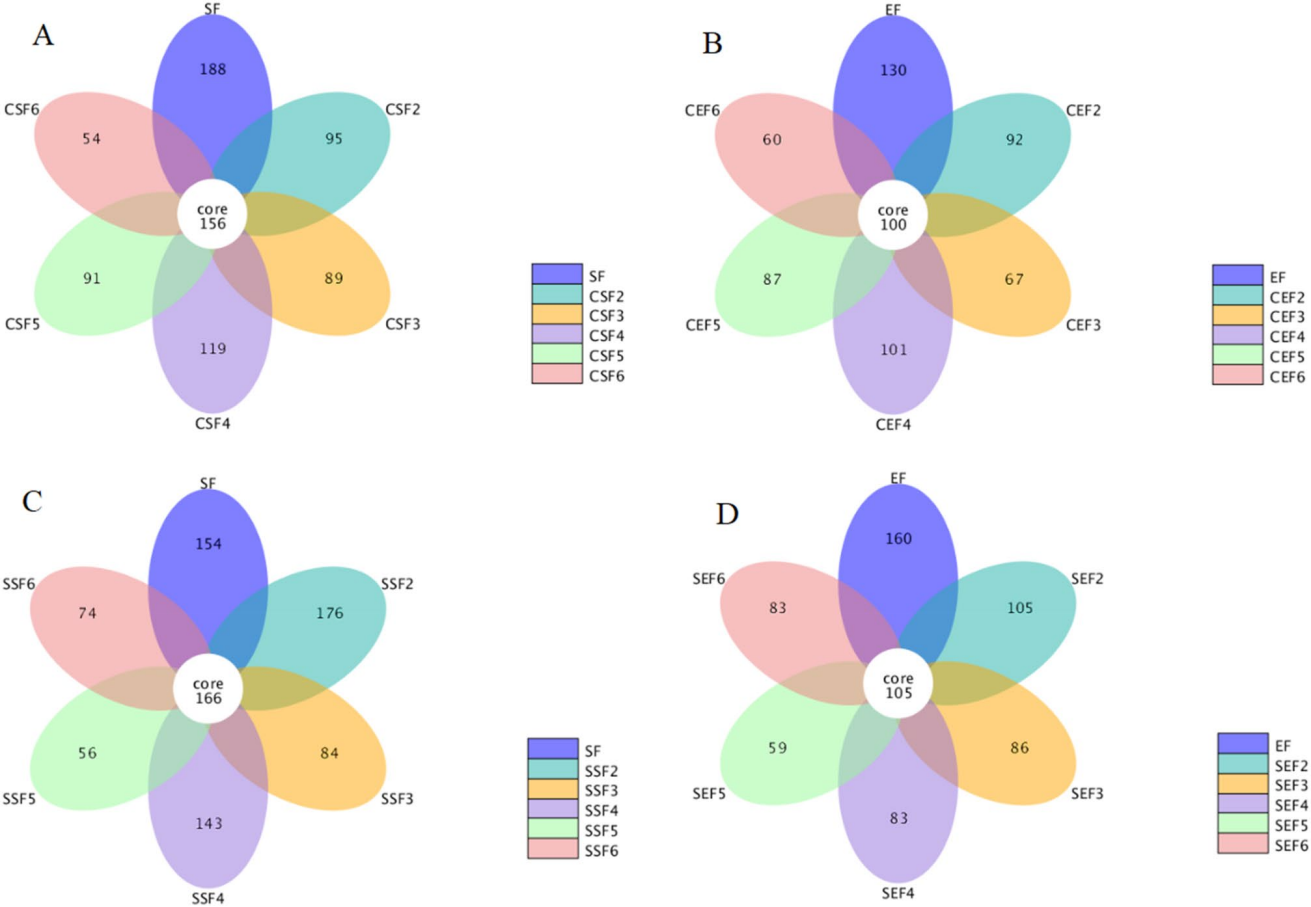
Petal diagrams of OTUs of fungi in samples flue-cured by using conventional procedure and temperature- and humidity-controlled procedure in different sampling stages (SB: the surface fungi of fresh tobacco leaves; EB: endophytic fungi of fresh tobacco leaves; CSB: the surface fungi of tobacco leaves flue-cured using conventional procedure; CEB: endophytic fungi of tobacco leaves flue-cured using conventional procedure; SSB: the surface fungi of tobacco leaves flue-cured using temperature- and humidity-controlled procedure; SEB: endophytic fungi of tobacco leaves flue-cured using temperature and humidity-controlled procedure; 2–6 denote different sampling stages.

The result is displayed in Fig. 4. As shown in the figure, the core microbial communities flue-cured by conventional procedure and temperature- and humidity-controlled procedure showed basically coincident numbers. The latter was only 5–10 core microbial communities more than the former. Similar to the core bacterial communities, the number of core fungal communities presented no great difference in the flue-curing process.

### Analysis of relative abundances of species

According to the results of species annotations, the species of each sample or group with the maximum abundance ranking the top 10–30 at various classification levels were selected to generate the cumulative histogram of relative abundances of species. The species with a high relative abundance in various samples at different classification levels and their proportions can be intuitively found.

#### Analysis of relative abundances of bacteria in tobaccos

Based on the results of species annotations, the species of each sample or group with the maximum abundance ranking the top 10–30 at various classification levels were selected to generate the cumulative histogram of relative abundances of species. The species with a high relative abundance in various samples at different classification levels and their proportions can be visualized.

**Figure 5:**
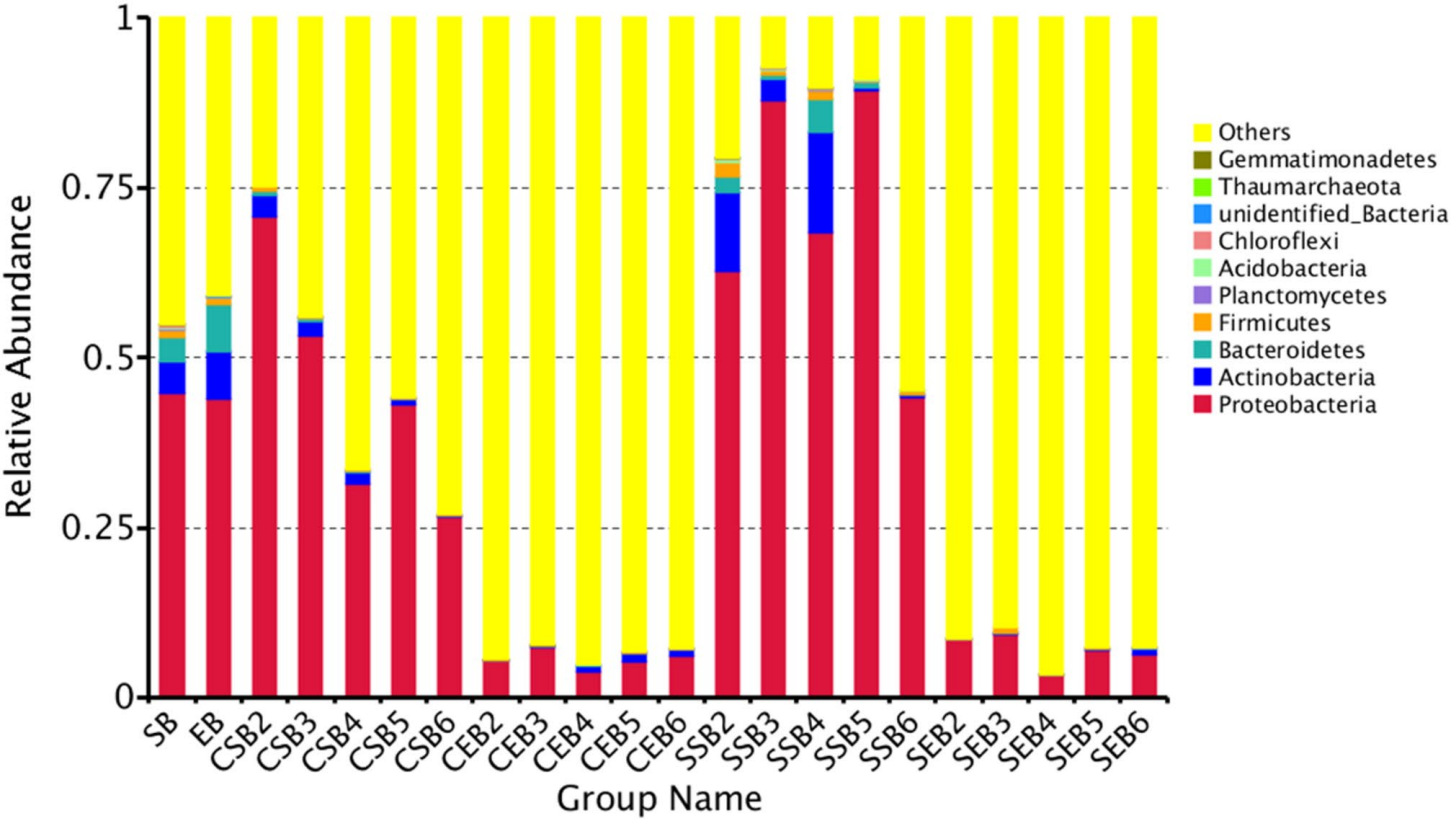
Histogram of relative abundances of species at the level of phylum.

As shown in Fig. 5, at the level of phylum, the bacteria in tobaccos mainly contained Proteobacteria, Actinobacteria, Bacteroidetes, Firmicutes, Planctomycetes, Acidobacteria, Chloroflexi, unidentified bacteria, Thaumarchaeota and Gemmatimonadetes. It can be seen that the surface and endophytic bacterial communities of fresh tobacco leaves slightly differed at the level of phylum. Proteobacteria showed the largest content, followed by Actinobacteria and Bacteroidetes, and the contents of the other bacterial phyla were relatively low. By using conventional flue-curing procedure, the bacterial diversity on the surface of tobacco leaves progressively declined as the flue-curing continued, and the relative content of Proteobacteria rose at first and then reduced; the reduction amplitude of Actinobacteria was relatively stable in the flue-curing process while that of Bacteroidetes was relatively large. For the dry-ball temperature set and wet-ball temperature degradation flue-curing procedure, as the flue-curing proceeded, the relative content of Proteobacteria gradually increased and it did not greatly reduce until reaching the last flue-curing stage. However, its relative content was not significantly different from that in fresh tobacco leaves; similar to Proteobacteria, the relative contents of both Actinobacteria and Bacteroidetes also grew at first and then decreased; in terms of endophytic bacteria of tobaccos, as the flue-curing process continued, the relative contents of Proteobacteria and the other main bacterial communities rapidly dropped while those of the other communities sharply increased.

**Figure 6:**
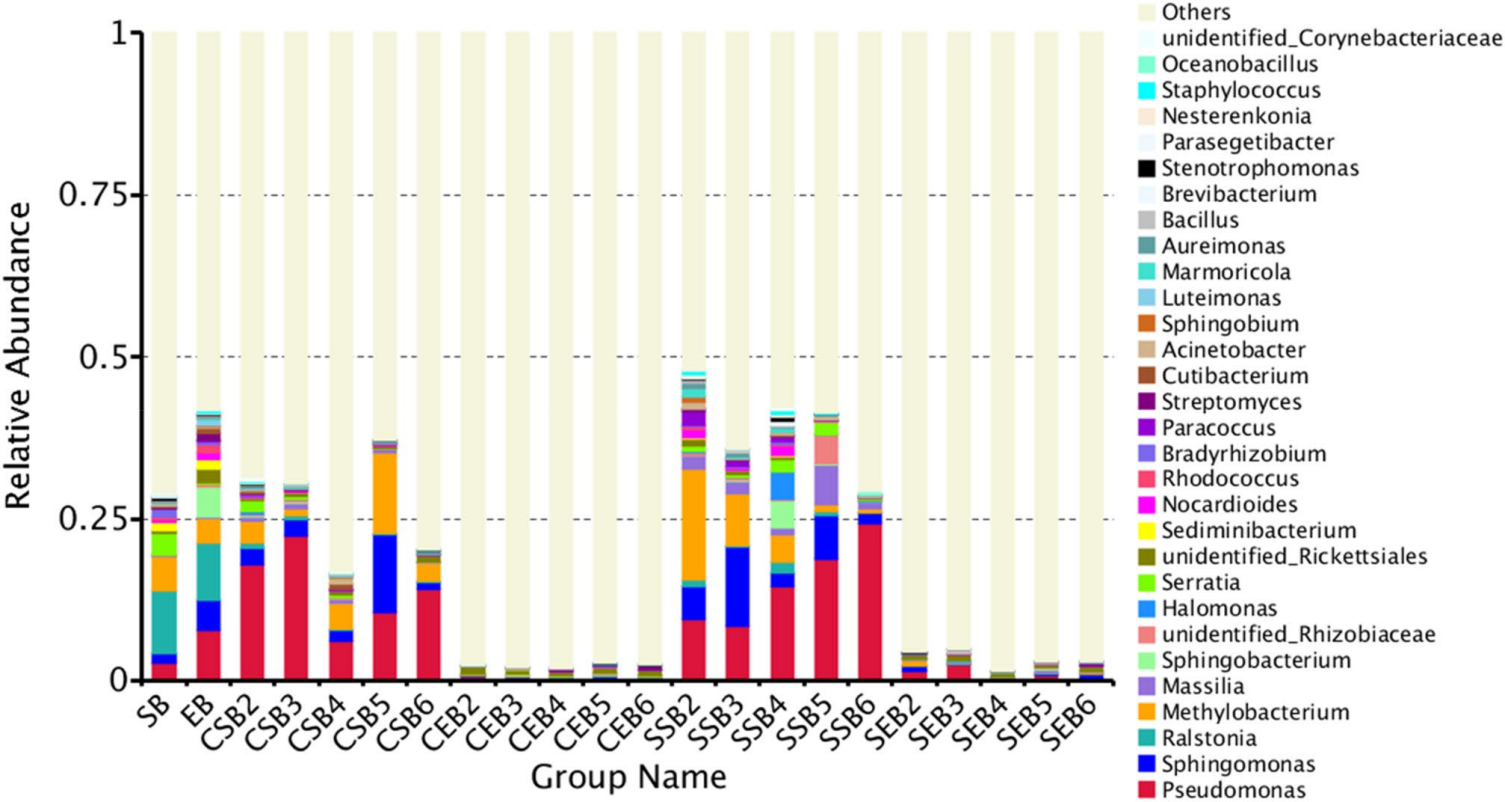
Column chart of species relative abundance at genus level.

As shown in Fig. 6, at the level of genus, the main dominant bacterial communities in endophytic bacteria of fresh tobacco leaves included *Pseudomonas*, *Sphingomonas*, *Ralstonia*, *Methylobacterium*, *Massilia*, *Sphingobacterium*, *Rhizobium*, *Halomonas*, *Serratia* and *Rickettsia*.

When employing conventional flue-curing procedure, the bacterial communities on the surface of tobaccos were relatively marginally changed at the level of genus while the endophytic bacteria varied remarkably. As the flue-curing process proceeded, the relative content of 30 main endophytic bacterial communities found before the flue-curing had dropped to 2% even in the early flue-curing stage (35 ℃). In comparison, the relative content of the bacterial communities in the first two flue-curing stages under dry-ball temperature set and wet-ball temperature degradation flue-curing procedure was higher.

Under conventional procedure, the relative abundance of *Pseudomonas* on the surface of tobacco leaves increased at first and then decreased, so did that of *Sphingomonas*. Although no signs of *Ralstonia* solanacearum were visualized on the surface of the sampled tobacco leaf samples, *Ralstonia* was found in the analysis of bacterial communities. With the ongoing flue-curing process, the relative content of *Ralstonia* rapidly reduced; the relative content of *Methylobacterium* on the surface of fresh tobacco leaves declined to some extent in the flue-curing process, and accounted for a large proportion in bacterial communities on the surface of flue-cured tobacco leaves. The relative contents of the other main bacterial communities were all progressively lowered basically.

When implementing dry-ball temperature set and wet-ball temperature degradation flue-curing procedure, the relative contents of the main bacterial communities in the early flue-curing stage were higher than those in fresh tobacco leaves. The relative contents of them marginally differed from those in fresh tobacco leaves even though flue-curing process was ended; the relative content of *Pseudomonas* gradually increased in the flue-curing process. By contrast, the relative contents of *Sphingomonas* and *Methylobacterium* both grew at first and then declined. The relative contents of the other main bacterial communities relatively slowly varied in the flue-curing process and they did not greatly decrease until the flue-curing process was ended. Moreover, the relative contents of some bacterial genera, including *Sphingobacterium* and *Rickettsia*, had remarkably dropped in the early flue-curing stage.

#### Analysis of relative abundances of fungi in tobaccos

**Figure 7:**
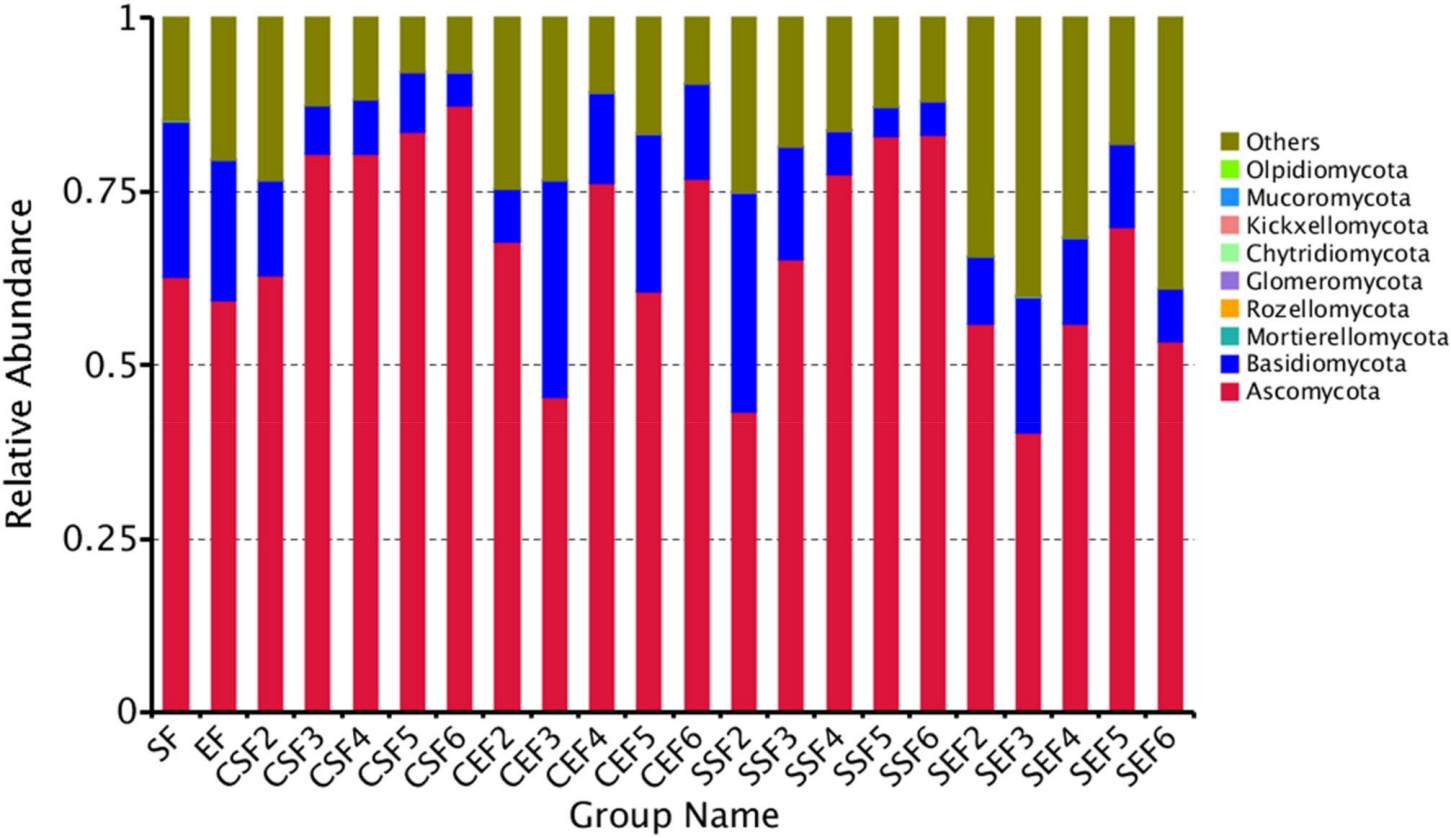
Histogram of relative abundances of species at the level of phylum.

As shown in Fig. 7, at the level of phylum, fungi in tobaccos mainly covered Ascomycota, Basidiomycota, Mortierellomycota, Rozellomycota, Glomeromycota, Chytridiomycota, Kickxellomycota, Mucoromycota and Olpidiomycota. It can be seen from the figure that fungi in tobaccos mainly included Ascomycota and Basidiomycota; the other fungi (phylum) took up a relatively low proportion.

When being flue-cured by using conventional procedure, the relative abundance of Ascomycota on the surface of tobacco leaves gradually increased while those of Basidiomycotaand the other fungal phyla gradually decreased with the ongoing flue-curing process; under temperature- and humidity-controlled flue-curing, the evolution law of fungal communities was similar to that using conventional procedure at the level of phylum. To be specific, a trend was shown that the relative abundance of Ascomycota gradually rose while those of Basidiomycota and the other fungal phyla were lowered, which was basically similar to that under conventional flue-curing procedure.

Their proportions were higher than those on the surface of tobacco leaves.The change amplitude of the endophytic fungi of tobacco leaves was less significant than that of fungi on the surface of tobacco leaves. Either under conventional flue-curing or temperature- and humidity-controlled flue-curing, the relative abundance of Ascomycota basically increased at first and then declined while that of Basidiomycota reduced at first, then grew and finally dropped.

**Figure 8:**
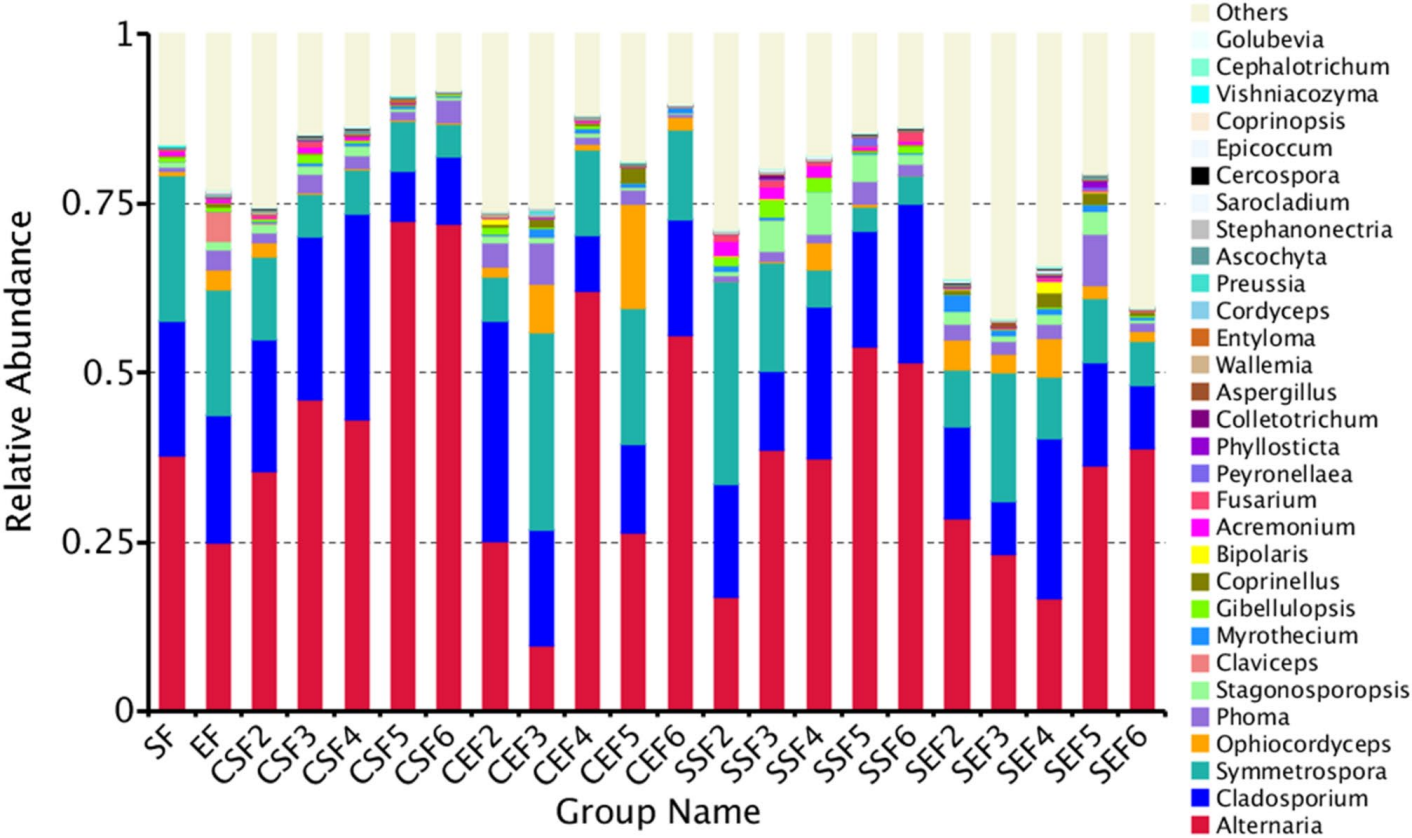
Histogram of relative abundances of species at the level of genus.

As shown in Fig. 8, the change trends of community compositions under conventional flue-curing and temperature- and humidity-controlled flue-curing at the level of genus were similar to those at the level of phylum. There was a great difference only in the relative contents of fungal communities. In terms of fungi on the surface of tobacco leaves, the relative content of *Alternaria* under conventional flue-curing greatly increased at 38.5 ℃ and 54 ℃, with increases at the same time points under temperature- and humidity-controlled flue-curing; however, the growth amplitude of the relative content was less significant than that under conventional flue-curing. *Cladosporium* was another main fungal community and its relative content slowly decreased in the later stage of temperature- and humidity-controlled flue-curing. The relative content of *Symmetrospora* progressively decreased when using conventional flue-curing procedure while its reduction rate slowed down under temperature- and humidity-controlled flue-curing. The relative content of *Ophiocordyceps* on the surface of tobacco leaves was relatively low and it both gradually reduced when using the two flue-curing technologies. Moreover, the relative contents of the other fungal genera also progressively declined as the flue-curing proceeded.

As for changes of endophytic fungi of tobaccos when using the two flue-curing technologies, the relative content of *Alternaria* under conventional flue-curing was higher than that under temperature- and humidity-controlled flue-curing. The result can be found even though the flue-curing process was ended. The relative content of *Cladosporium* marginally varied under conventional flue-curing and greatly increased at 35 ℃. After completing the flue-curing process, the relative content did not significantly differ from the value in fresh tobacco leaves. However, for dry-ball temperature set and wet-ball temperature degradation flue-curing procedure, the relative content of *Cladosporium* progressively reduced on the whole and the value after ending the flue-curing process was only about half of that in fresh tobacco leaves. The relative content of *Symmetrospora* showed a same change trend with *Cladosporium* under conventional flue-curing and temperature- and humidity-controlled flue-curing. Additionally, the reduction rate of the relative content of *Symmetrospora* was higher than that of *Cladosporium* under temperature- and humidity-controlled flue-curing. Relative to fungal communities on the surface of tobaccos, although the relative contents of the other endophytic fungal genera gradually dropped with the flue-curing.

### Clustered heat maps of species abundances

According to species annotations and abundances of all samples at the level of genus, genera whose abundances ranked the top 35 were selected. Subsequently, based on the abundances of these genera in each sample, a heat map is drawn by conducting clustering from the two aspects: i.e. species and samples. By doing so, it is convenient to ascertain a species with a high abundance or low content and the sample from which it is found.

#### Clustered heat map of species abundances of bacteria in tobaccos

**Figure 9:**
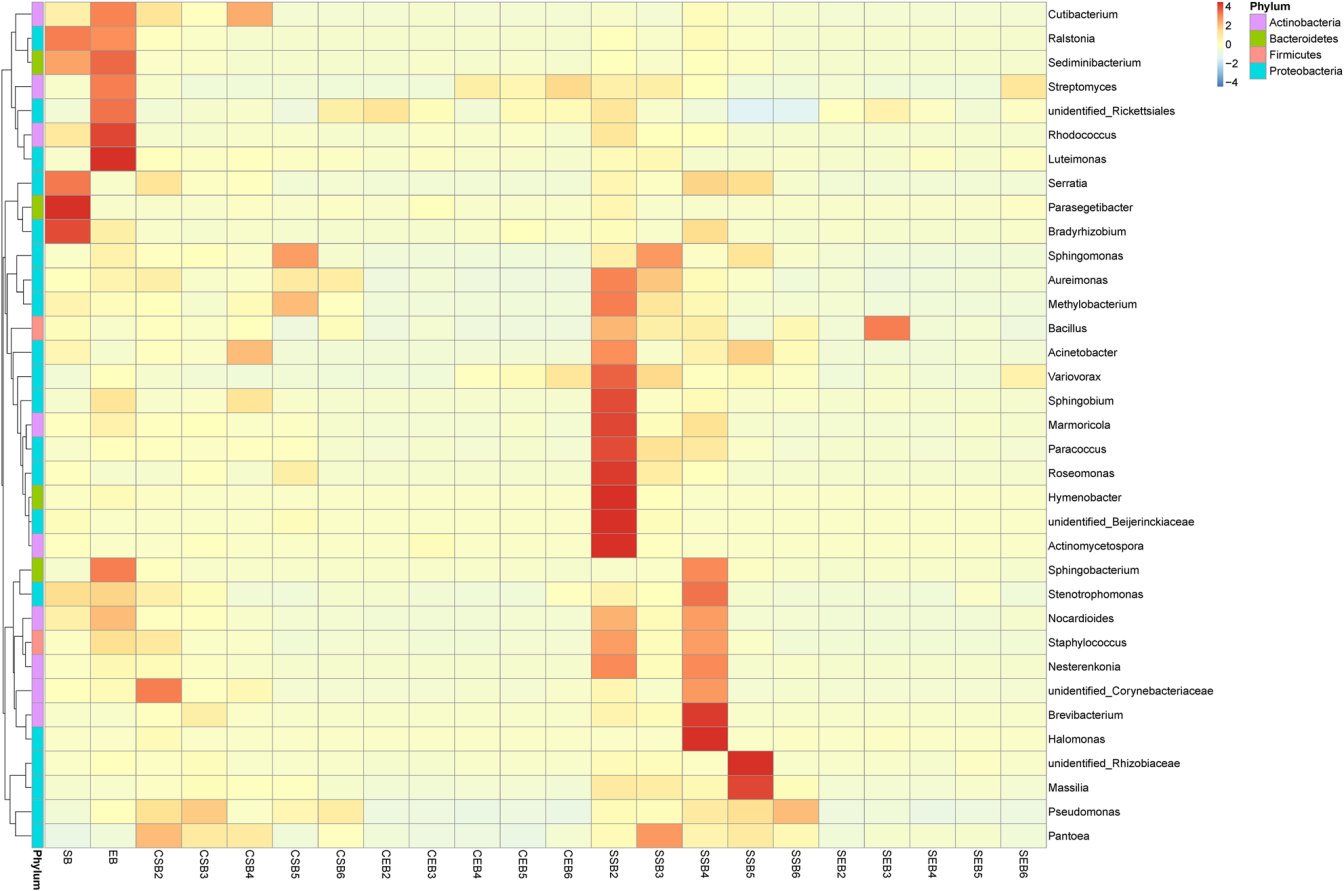
Clustered heat map of species abundances at the level of genus.

The result is displayed in Fig. 9. It can be seen from the clustered heat map that bacterial communities mainly reside on the surface and in the interior of the fresh tobacco leaves at first. As the flue-curing proceeded, the contents of the main bacterial communities were changed to some extent: the main bacterial communities reduced in the content and the tobacco leaves became light in color.

#### Clustered heat map of species abundance of fungi in tobaccos

**Figure 10:**
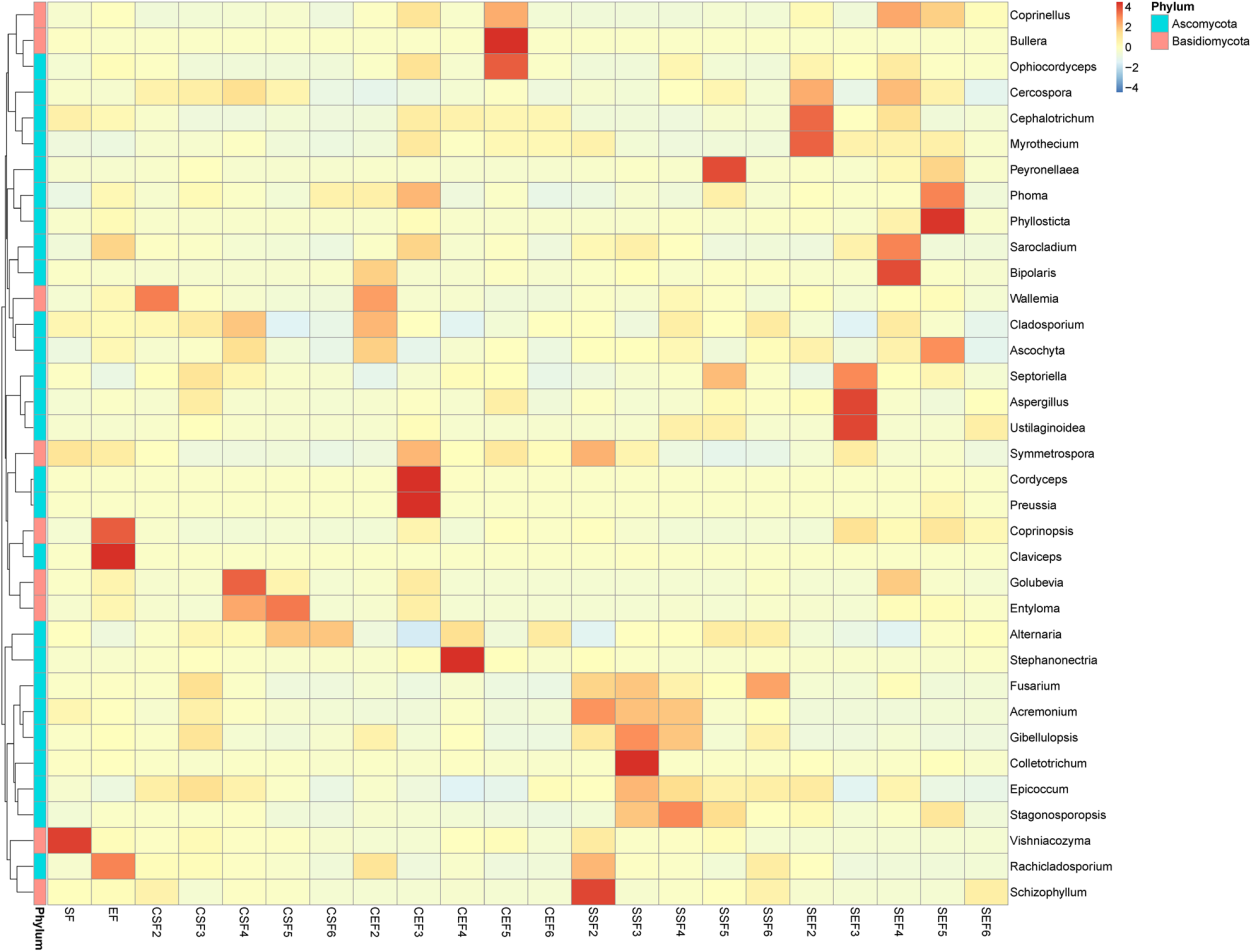
Clustered heat map of species abundances at the level of genus.

According to species annotations and abundances of all samples at the level of genus, the genera whose abundances ranked the top 35 were selected. Based on the abundances of these genera in each sample, a heat map is drawn by conducting clustering from the two aspects, i.e. species and samples. It can be found from Fig. 10 that the distribution of fungi on the surface of tobaccos greatly differed from that of endophytic fungi. Moreover, the distribution of materials also presented a great difference when using the two flue-curing technologies.

### Correlation with environmental factors

#### Correlation of bacteria with environmental factors

**Figure 11:**
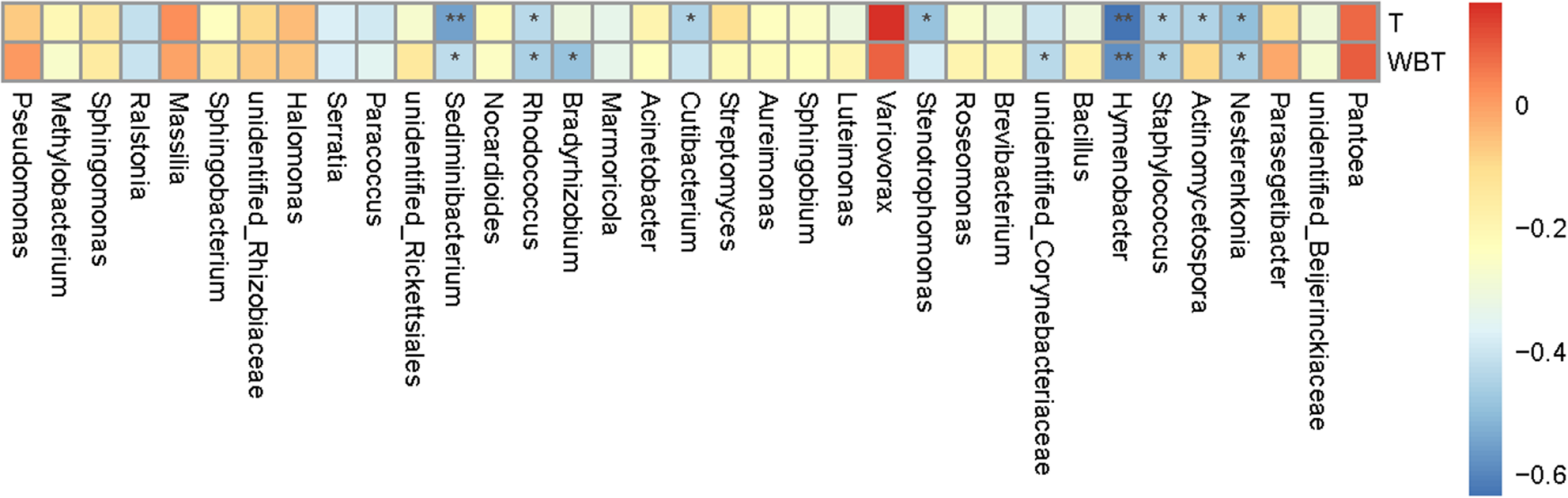
Heat map of correlation with environmental factors at the level of genus.

Temperature and humidity were mainly controlled in the flue-curing process of tobacco leaves; and the main environmental factors involved dry- and wet-bulb temperatures. It can be seen from Fig. 11 that *Pantoea*, *Nesterenkonia*, *Staphylococcus*, *Variovorax*, *Chryseomonas*, *Rhodococcus*, *Paracoccus*, *Massilia*, *Serratia*, *Ralstonia* and *Pseudomonas* were more likely to be affected by temperature and humidity. *Pantoea* and *Variovorax* exhibited a positive correlation with temperature and humidity; *Nesterenkonia*, *Staphylococcus*, *Chryseomonas*, *Rhodococcus*, *Paracoccus*, *Serratia* and *Ralstonia* presented a negative correlation with temperature and humidity. *Actinomycetospora* were negatively correlated with the dry-bulb temperature while positively correlated with the wet-bulb temperature; *Stenotrophomonas*, *Cutibacterium* and *Sediminibacterium* were all negatively correlated with both dry- and wet-bulb temperatures while they presented a higher negative correlation with the dry-bulb temperature.

#### Correlation of fungi with environmental factors

**Figure 12:**
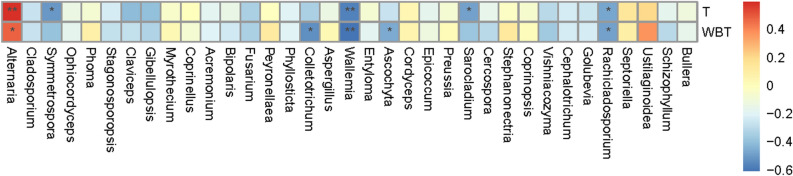
Heat map of correlation with environmental factors at the level of genus.

Similar to the analysis method of environmental factors of bacteria, the correlation between the fungi in tobaccos and environmental factors is displayed in Fig. 12. Temperature and humidity were mainly controlled in the flue-curing process of tobacco leaves; the main environmental factors were dry- and wet-bulb temperatures. As shown in the figure, different from the correlation of bacterial communities in tobaccos with environmental factors, the majority of fungal genera in tobaccos presented a negative correlation with temperature and humidity, for example, *Rachicladosporium*, *Vishniacozyma*, *Symmetrospora*, *Sarocladium*, *Ascochyta*, *Wallemia*, *Colletotrichum*, *Fusarium*, *Claviceps*, *Cladosporium*, etc. A small number of fungal genera (such as *Ustilaginoidea*, *Septoriella* and *Alternaria*) were positively correlated with temperature and humidity.

### Function prediction of bacterial communities in tobaccos

According to the annotation result in the database, the functions with the maximum abundance ranking the top 10 in each sample or group at various layers of annotations were selected to generate the cumulative histogram of relative abundances of functions. Thus, it is convenient to check the functions with a high relative abundance in various samples at different layers of annotations and their proportions.

**Figure 13:**
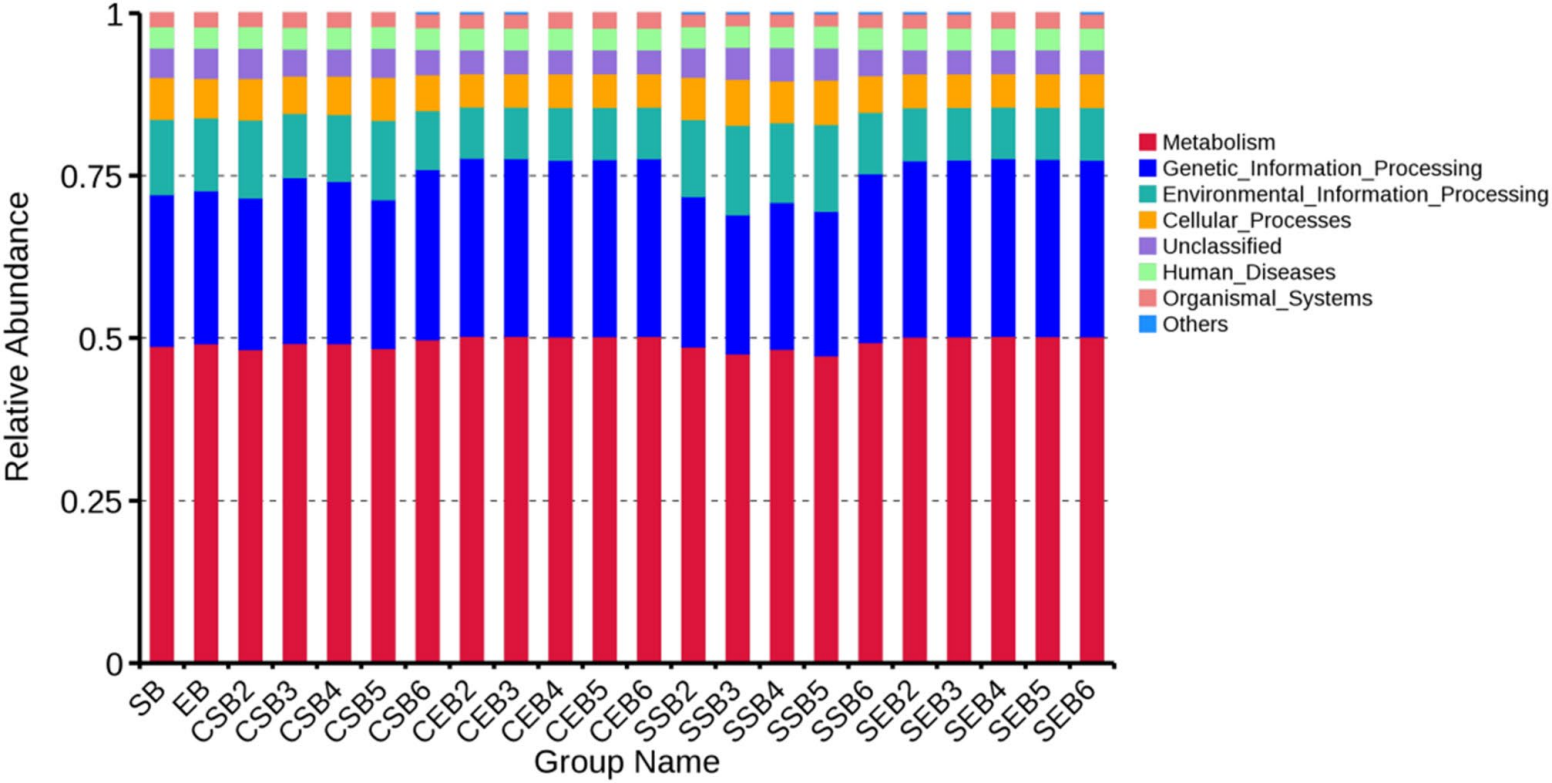
Relative abundances of function annotations of bacterial communities in tobaccos.

As shown in Fig. 13, bacterial communities with a half of relative abundances participated in the metabolism and a quarter of bacterial communities took part in the genetic information processing; the rest was engaged in the cellular processes and organismal systems and some bacterial communities were implicated to human diseases.

### Function prediction of fungal communities in tobaccos

**Figure 14:**
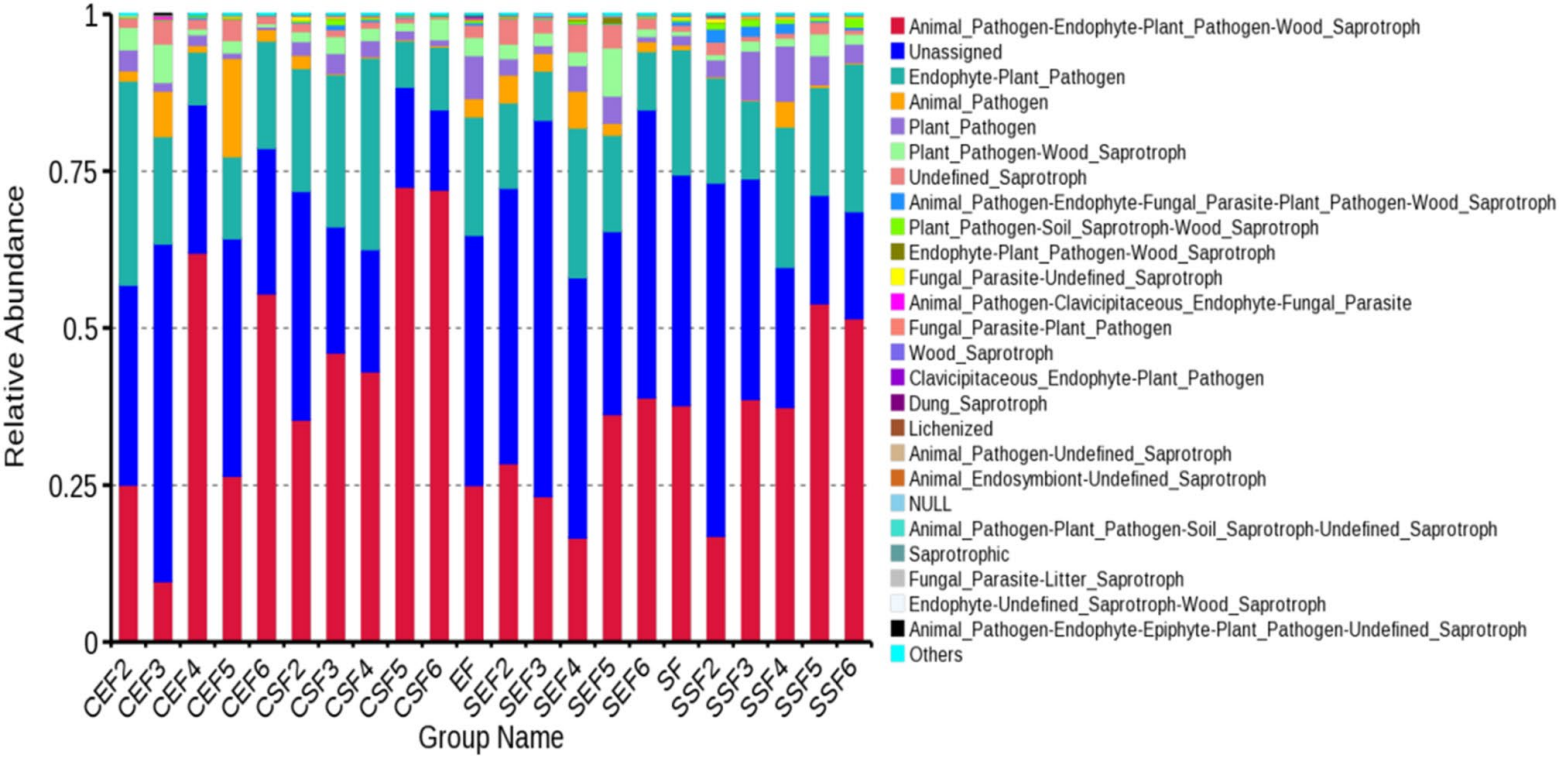
Relative abundances of function annotations of fungal communities in tobaccos.

Based on amplificon analysis of 16S or ITS, the species classification and abundances of fungi present in the environment can be attained. In many cases, people will also concern what role these species found in the environment play in the ecological environment. By applying FunGuild tool, it is feasible to attain the ecological functions of corresponding fungi based on the species classification of fungi. It can be seen from the Fig. 14 that the fungal communities in tobaccos delivered relatively abundant functions, in which the three fungal communities with the highest relative abundances separately showed the following functions: plant saprophytes, plant pathogens and undefined functional fungal communities; the rest of fungal communities participated in plant parasitism,soil-borne plant pathogen, lichenization, dung saprotroph, etc.

## Discussion

### Comparison of sampling methods for microbes in tobacco leaves

As for the surface wiping and washing methods for sampling microbes on the surface of tobacco leaves, the latter is more suitable. The reason is that due to the large amount of oil on the surface of tobacco leaves, the swabs for sampling microbes are found to be obviously blackened in the sampling process when using surface wiping method^27^. As a result, the extracted microbe samples are likely to be greatly affected by impurities, which is unfavorable for subsequent DNA extraction^28^.

### OTU clustering analysis of microbes in tobacco leaves

The number of core bacterial communities in tobacco leaves under conventional flue-curing was basically consistent with that under temperature- and humidity-controlled flue-curing. Although there was a certain difference of temperature and humidity in various stages when using conventional and temperature- and humidity-controlled flue-curing technologies, the final temperatures were coincident. It was the main reason why the core bacterial communities under the two procedure nearly showed no difference. The final temperature under temperature- and humidity-controlled flue-curing was the same as that under conventional flue-curing; despite this, owing to the significantly different control over temperature and humidity in the two flue-curing processes, the numbers of OTUs presented a great difference in the flue-curing process even if sampling was performed at a same dry-bulb temperature. Dry-ball temperature set and wet -ball temperature degradation flue-curing procedure can favorably maintain the diversity of bacteria on the surface of tobacco leaves before reaching 48 ℃. By analyzing the distribution of OTUs of surface and endophytic bacteria of tobacco leaves, it can be seen that the abundance of the surface bacteria was remarkably higher than that of endophytic bacteria in the flue-curing process; this indicated that the endophytic bacteria of tobaccos were more sensitive to temperature and humidity and the reduction rate of OTUs of endophytic bacteria was obviously larger than that of surface bacteria in the flue-curing process. As the flue-curing process started, the numbers of OTUs of surface and endophytic bacteria of tobaccos both dramatically reduced. The bacteria on the surface of tobaccos were sensitive to temperature and only a small number of communities could be tolerant to a high temperature^29^.

Similar to core bacterial communities, the numbers of core fungal communities presented no great difference in the flue-curing processes. The possible reason was that both differences of temperatures and humidities in the two flue-curing processes were relatively consistent, with a slight difference. Although the final temperature and humidity under temperature- and humidity-controlled flue-curing were the same as those under conventional flue-curing, the heating rates exhibited a certain difference. Therefore, the main fungal communities showed a great difference in the flue-curing processes. Relative to bacteria in tobaccos, the difference of the distributions of the surface and endophytic fungi of tobacco leaves was less significant, indicating that the endophytic fungal communities of tobaccos were more abundant^30^. With the ongoing flue-curing process, the OTUs of surface and endophytic fungi of tobacco leaves gradually declined; however, they did not sharply reduce, which differed from bacteria. It implied that the adaptability of fungi in tobaccos was superior to that of bacteria in the flue-curing process^4^. In terms of the number of OTUs, the OTUs of fungi in tobaccos were remarkably more than those of bacteria, implying that fungi accounted for a large proportion in microbial communities in tobaccos. The possible reason was that fungi presented a harmonic symbiosis with tobaccos^31–33^.

### The effect of different flue-curing procedure on microbial diversity of tobaccos

Only the diversity of bacterial communities on the surface of tobacco leaves in the second stage under temperature- and humidity-controlled flue-curing was greatly higher than that under conventional flue-curing; however, in the other stages, the two flue-curing procedure showed no great difference in influences on the diversity of microbial communities in tobaccos, and the numbers of microbial communities under the two flue-curing procedure basically showed a same change trend. Compared with surface bacteria, the flue-curing proceduresignifican the diversity of endophytic bacteria and the core OTUs number was only half of surface bacterial. As the flue-curing procedure conducts, the diversity of endophytic bacterial falls sharply and the OTUs numbere was only one fifth of freshe tobacco leaves.At the level of genus, the main bacterial communities in tobaccos included *Pseudomonas*, *Sphingomonas*, *Ralstonia*, *Methylobacterium*, *Massilia*, *Sphingobacterium*, *Rhizobium*, *Halomonas*, *Serratia* and *Rickettsia*. Relative to conventional flue-curing procedure, the surface and endophytic bacteria of tobacco leaves showed higher diversity under temperature- and humidity-controlled flue-curing. This suggested that dry-ball temperature set and wet-ball temperature degradation flue-curing procedure can favorably maintain the diversity of microbial communities in tobaccos.

The diversity of fungi was more better than bacteria. The core OTUS number of fungi was four times higher than bacteria. After the flur-curing procedure, more than sixty percent OTUs number can be maintained and the change of OTUs number was not dramatically as the OTUs number of bacteria.In terms of conventional procedure, the relative contents of *Pseudomonas* (endophytic), *Sphingomonas* (surface), *Methylobacterium* (surface) and *Massilia* (surface) were obviously high in the early flue-curing stage. However, *Pseudomonas* (surface) delivered an obviously low relative content and there was a slight difference in relative contents of the rest of main bacterial genera. No great difference was found in terms of the effects of conventional and temperature- and humidity-controlled flue-curing procedure on the diversity of fungal communities in tobaccos; the fungal communities were obviously more diverse, and had a number of OTUs more than twice, of the bacteria. At the level of genus, the main fungal communities included *Alternaria*, *Cladosporium*, *Symmetrospora*, *Ophiocordyceps*, *Phoma*, *Stagonosporopsis*, *Claviceps*, *Myrothecium*, *Gibellulopsis* and *Coprinopsis*. Relative to conventional flue-curing, the relative contents of *Alternaria* (surface) and *Cladosporium* (endophytic) were obviously lower while that of *Symmetrospora* (surface) was significantly higher under temperature- and humidity-controlled flue-curing. Besides, the relative contents of the other main fungal genera showed a marginal difference.

### Dynamic change of compositions of microbial communities in tobaccos treated using flue-curing technologies

Under conventional flue-curing, the bacterial communities on the surface of tobaccos were slightly changed while the endophytic bacterial communities greatly varied at the level of genus. The relative content of 30 main endophytic bacterial communities of tobacco leaves before the flue-curing was 32%. However, as the flue-curing process proceeded, their relative content had dropped to 2% even in the early flue-curing stage (35 ℃), which only took up one tenth of the previous. It indicated that the endophytic bacteria of tobaccos at the level of genus were quite sensitive to flue-curing technologies. The relative content (being up to 4%) of the bacterial communities treated by using dry-ball temperature set and wet-ball temperature degradation flue-curing procedure was higher than that treated by applying conventional procedure in the first two flue-curing stages (35 ℃ and 38.5 ℃). Subsequently, it declined to be about 2%, implying that dry-ball temperature set and wet-ball temperature degradation flue-curing procedure was better than conventional procedure to some extent in terms of maintaining the bacterial diversity. The relative content of the main bacterial communities in the early stage of temperature- and humidity-controlled flue-curing was higher than that of fresh tobacco leaves. No great difference was found between the two parties even if flue-curing process was completed; moreover, the dry-ball temperature set and wet-ball temperature degradation flue-curing procedure was also superior to conventional flue-curing procedure in maintaining the abundance of bacterial communities. By analyzing the distribution of bacterial communities, it can be seen that dry-ball temperature set and wet-ball temperature degradation flue-curing procedure was beneficial for keeping the bacterial diversity, and the main bacterial communities also accounted for a large proportion; the relative content of endophytic bacteria was remarkably affected by the flue-curing process: the content of the main endophytic bacterial communities in the early flue-curing stage only took up one tenth of the initial content.

In terms of fungi on the surface of tobaccos treated by utilizing conventional flue-curing procedure, the relative content of Ascomycota gradually rose while that of Basidiomycota progressively dropped with the ongoing flue-curing process. The evolution law of fungal communities at the level of phylum when using dry-ball temperature set and wet-ball temperature degradation flue-curing procedure was similar to that under conventional procedure. It also showed the result that the relative content of Ascomycota gradually grew while those of Basidiomycota and the other fungal phyla progressively decreased. After ending the flue-curing process, the relative contents of Ascomycota, Basidiomycota and the other fungal phyla were 83%, 5% and 12%, respectively, which were basically similar to the results under conventional procedure. The change amplitude of endophytic fungi of tobacco leaves was less significant than that of surface fungi. Either using the conventional or the temperature- and humidity-controlled flue-curing technologies, the relative content of Ascomycota basically increased at first and then declined while that of Basidiomycota reduced at first, then rose and finally declined again. It implied, at the level of phylum, that the two flue-curing procedure showed different effects on the distribution of fungal communities of tobaccos in the flue-curing process. In terms of the relative content, dry-ball temperature set and wet-ball temperature degradation flue-curing procedure was favorable for the survival of the other fungal phyla while it did harm to the survival of Ascomycota and Basidiomycota. The change trends of compositions of fungal communities treated by using conventional and temperature- and humidity-controlled flue-curing procedure at the level of genus were similar to those at the level of phylum. The difference resided in the significant difference in the relative contents of fungal communities. On the whole, the relative contents of fungal genera slowly reduced under temperature- and humidity-controlled flue-curing.

### Correlation with environmental factors and function prediction of communities

It can be found from the correlation with environmental factors that the majority of bacterial communities were all basically negatively correlated with dry- and wet-bulb temperatures. It revealed that dry- and wet-bulb temperatures could lower the abundance of bacterial communities. The flue-curing promoted the material transformation and metabolism of tobacco leaves, in which macromolecules were transformed into small molecule compounds^34,35^. In terms of function prediction of bacteria in tobaccos, the function distributions of either the surface or endophytic bacteria of tobacco leaves were all similar either under conventional or temperature- and humidity-controlled flue-curing. This suggested that the functions of the bacteria were basically consistent when using the two flue-curing technologies, both mainly focusing on transformation of compounds, thus promoting the transformation of the quality of tobacco leaves^36^.

Different from the correlation of bacterial communities in tobaccos, the majority of fungal genera in tobaccos presented a negative correlation with temperature and humidity. Only a small number of fungal genera were positively correlated with temperature and humidity. Compared with bacteria in tobaccos, the functions of fungi in tobaccos remain to be further explored. The related fungal communities mainly function in plant parasitism^37^; however, the functions of fungal communities in tobaccos fail to be analyzed from the perspective of the tobacco quality.

In present study, the bacterial and fungal community diversity were significant affected by the flue-curing procedure and the microorganisms have a relativity of substance transformation in tobacco. The succession of microbial community can guides the improvement of flue-curing technology.

## Conclusions

1. Compared with surface wiping method, the washing method was more applicable for sampling the microbes on the surface of tobacco leaves.
2. Conventional and temperature- and humidity-controlled flue-curing procedure presented a similar effect on core microbial communities of tobaccos; however, the latter was more favorable for maintaining the microbial diversity of tobaccos. This conclusion also provides some technical support and theoretical basis for the promotion of conventional flue-curing procedure.
3. Compared with bacteria of tobaccos, the succession law of fungal communities in tobaccos was relatively steady. Moreover, the number of OTUs of fungal communities in tobaccos was greatly higher than that of bacterial communities. The compositions of bacterial communities were relatively simple and several dominant communities accounted for quite a high proportion; however, there were many dominant fungal communities in tobaccos. It indicated that the diversity of fungi was superior to bacteria. This indicated that the fungi of tobaccos have a better applicability during the flue-curing process.
4. By analyzing environmental factors, temperature and humidity delivered a negative correlation with fungi of tobaccos in the flue-curing process; however, the correlation coefficient was low; the fungal communities of tobaccos were greatly negatively correlated with temperature and humidity. The results indicated that the higher flue-curing temperature was is detrimental to the survival of microorganisms, especially for fungi.
5. The functions of bacterial communities in tobaccos matched with the material transformation law of tobaccos, playing an important role in the flue-curing process. However, the functions of fungi remain unclear so the functions of fungi in the flue-curing process have not been clarified. For future research, the researchers should pay more attentions on the functional microorganisms of tobacco and enhance the function to improce the quality of flue-cured tobacco.
